# Comparison of the safety and efficacy of anterior ‘skip’ corpectomy versus posterior decompression in the treatment of cervical spondylotic myelopathy

**DOI:** 10.1186/s13018-014-0063-x

**Published:** 2014-09-25

**Authors:** Lie Qian, Jiang Shao, Zude Liu, Liming Cheng, Zhili Zeng, Yongwei Jia, Xinfeng Li, Hantao Wang

**Affiliations:** Department of Orthopedics, Renji Hospital, Shanghai Jiao Tong University School of Medicine, No. 1630 Dongfang Road, Shanghai, 200127 China; Department of Orthopedics, Xinhua Hospital, Shanghai Jiao Tong University School of Medicine, Shanghai, 200127 China; Department of Orthopedics, Tongji Hospital, Shanghai, 200127 China

**Keywords:** Cervical spondylotic myelopathy, ‘Skip’ corpectomy, Posterior decompression

## Abstract

**Background:**

The aim of this study was to compare the therapeutic effects of anterior ‘skip’ corpectomy with posterior decompression for treating four-level cervical spondylotic myelopathy.

**Methods:**

Operation time and blood loss during the operation for the anterior and posterior approach groups were recorded. Patients were examined with cervical lateral radiography before and after the operation to measure Cobb's angle and postoperatively to monitor bony fusion. Surgery-, instrumentation-, and graft-related complications were assessed and recorded.

**Results:**

The surgical aspects of both anterior ‘skip’ corpectomy and posterior decompression went smoothly, with mean durations of 2.5 and 2.1 h, respectively, and mean blood loss volumes of 250 and 380 mL, respectively. In the anterior approach group, the complications included axial pain in five cases and transient hoarseness in two. Radiography revealed titanium mesh subsidence in two cases and plate or screw dislodgement in one case. In the posterior approach group, C5 nerve root palsy was present in 2 patients, axial pain in 15, and cerebrospinal fluid leakage in 3. The mean Japanese Orthopaedic Association scores showed that the recovery rate was significantly higher in the anterior approach group than in the posterior approach group (*p* < 0.05).

**Conclusions:**

‘Skip’ corpectomy has comparable safety and better efficacy than posterior decompression in the treatment of four-level cervical spondylotic myelopathy.

## Introduction

Cervical spondylotic myelopathy (CSM) is the most common degenerative disease in patients over 55 years of age. Narrowing of the spinal canal with age leads to compression of the spinal cord, causing a variety of symptoms (e.g., pain, numbness, and weakness) that, in turn, result in disabilities that negatively affect human health [1]. When the clinical symptoms are severe and progressive, surgery may be required to widen the spinal canal to alleviate spinal cord compression. Although research on the diagnosis and treatment of these diseases has made progress, the optimal surgical approach for multilevel CSM remains controversial.

Indications for anterior cervical discetomy are restricted to some extent because of the limited surgical field. Additionally, complications are more likely to develop after multilevel corpectomy [2]. Thus, a posterior approach to three-level or multilevel CSM was proposed to avoid the complications associated with the anterior approach [3,4]. Based on biomechanical [5] and clinical research, several authors have recently suggested an approach that combines anterior and/or posterior decompression and fixation to treat multilevel CSM [6]. For four-level CSM, however, an increasing number of surgeons prefer ‘skip’ corpectomy and decompression [7,8]. It is a new technique to obtain optimum decompression and fixation for patients suffering from multilevel CSM and ossification of the posterior longitudinal ligament (OPLL). Yet, there has been no detailed comparative study comparing the anterior ‘skip’ corpectomy with a posterior approach for treating CSM with four-level spinal canal stenosis. We therefore conducted a retrospective review of 198 patients who suffered from CSM with four-level spinal canal stenosis and who underwent either (1) anterior ‘skip’ corpectomy with decompression, graft fusion and internal fixation or (2) a posterior approach operation. This study aimed to prove that skip corpectomy is at least as safe and efficacious as posterior decompression.

## Materials and methods

### General information

Between 2005 and 2009, a total of 336 patients underwent an operation for CSM with four-level spinal canal stenosis with or without OPLL. Of these 336 patients, 198 patients who underwent only anterior ‘skip’ corpectomy from 2005 to 2009 (anterior group) or only posterior spinal canal decompression and internal fixation from 2007 to 2009 (posterior group) with more than 2 years of follow-up were enrolled in this study. All of the patients suffered from C3 to C7 cervical cord compression. Among them, 43 patients (24 men and 19 women) with a mean age at operation of 65.0 years (range 48 to 79 years) underwent anterior ‘skip’ corpectomy. The other 155 patients (88 men and 67 women) with a mean age at operation of 65.4 years (range 49 to 81 years) underwent posterior spinal decompression and internal fixation. Before the operation, the patients were examined with cervical vertebral anteroposterior and lateral radiography, flexion-extension lateral radiography, computed tomography (CT), and magnetic resonance imaging (MRI) of the cervical vertebra. Cobb's angle (C2 to C7) was measured on radiographs. CT and MRI were used to define the lesion site (C3 to C7).

All patients gave informed consent prior to their inclusion in the study. All human studies were approved by the Renji Hospital Ethics Committee and were performed in accordance with ethical standards.

### Surgical techniques

**Figure 1:**
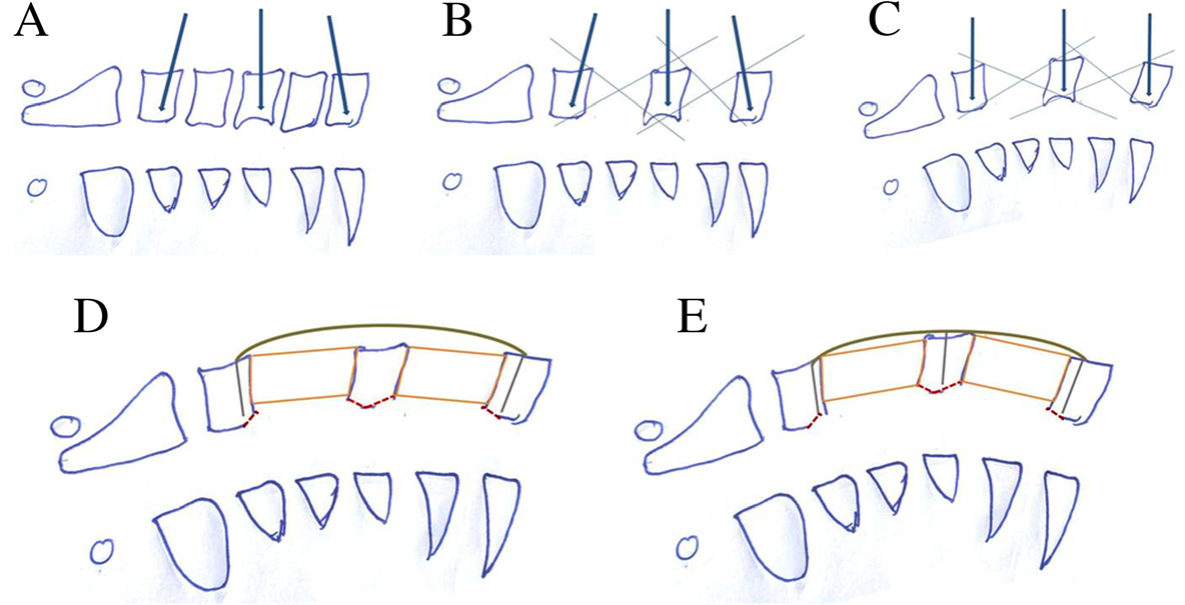
**Intraoperative decompression and restoration of the cervical curvature. (A)** One screw was perpendicular to the C5 vertebra. Screws for C3 and C7 vertebrae were inclined cephalad and caudally as required. **(B)** C4 and C6 corpectomy (relatively small visual fields). **(C)** After corpectomy, a distraction device was placed and distracted properly. Anterior vertebral bodies were correspondingly distracted. The cervical curvature was restored. With enlargement of the surgical visual fields, articles causing compression can be located posterior to the vertebrae and removed under direct vision. **(D)** Removal of articles causing compression, bone grafting, and proper placement of an already bent anterior cervical plate. C3 and C7 screws were placed first. **(E)** The C5 screw was placed last. The spine was lifted and repositioned to further restore the cervical curvature.

The anterior approach (anterior ‘skip’ corpectomy with decompression, graft fusion, and internal fixation) was performed under general anesthesia delivered by a nasal tracheal cannula. The patient was placed in the conventional posture for a cervical anterior approach: supine with a pillow placed under the shoulder and neck so there was slight hypokinesis of the head and a relaxed cervical area. A right anterior cervical oblique incision was made. C4 and C6 corpectomy and decompression of the posterosuperior and posteroinferior aspects of the C5 vertebra were then performed. We removed osteophytes and any other bodies compressing the spinal cord until it was fully decompressed. Titanium mesh and bone grafts were then implanted in C3 to C5 and C5 to C7. A properly pre-bent locking plate of suitable length was placed. Bicortical screws were fixed in C3 and C7 followed by a bicortical screw in C5. We then lifted and repositioned the spine to further restore the cervical curvature (Figure 1). Negative-pressure drainage was placed in the incision for 24 to 48 h.

The posterior approach was performed under general anesthesia with tracheal intubation. The patient was prone with the neck bent slightly forward. A posterior midline approach was used. Bicortical screw fixation was performed in the C3 to C7 lateral mass or vertebral pedicle. The C3 to C7 spinous process and laminae were removed to decompress the area. Facets were removed, and bone grafts were implanted into bilateral facet joints. Negative-pressure drainage was placed in the incision for 24 to 48 h.

Postoperatively for both procedures, conventional doses of a second-generation cephalosporin were prescribed for 2 days. Furosemide (20 mg i.v. injection) and dexamethasone (20 mg i.v. drip) were given daily. Furosemide was withdrawn, and the dose of dexamethasone was reduced to 10 mg 3 days later. Then, dexamethasone was withdrawn 2 days later. With a Philadelphia collar fixed, patients took part in off-bed activities and functional exercises on postoperative day 1. The Philadelphia collar was worn for 3 months by patients who underwent anterior approach surgery and for 2 weeks by those who underwent posterior approach surgery.

### Follow-up and evaluation method

Lateral radiography of cervical vertebrae was used to evaluate the recovery of the physiological curvature. Cervical lordosis was calculated with Cobb's angle, which was measured on the basis of the inferior edge of C2 to C7. The radian of the cervical vertebrae was defined as positive when the curvature trended forward and as negative when backward. Any breakage or displacement of the titanium mesh or locking plate was recorded, as was bone graft fusion. Anterior fusion was assessed using flexion-extension lateral radiography. Fusion was achieved when the differences of the distances between the spinous processes at the level of fusion in extension and flexion were less than 2 mm.

Recovery of neurological status was evaluated based on the Japanese Orthopaedic Association (JOA) scoring system. The recovery rate was calculated using preoperative and postoperative JOA scores and the following formula [9]:

$$ \left(\mathrm{Postop}.\;\mathrm{J}\mathrm{O}\mathrm{A}\;\mathrm{score}-\mathrm{Preop}.\;\mathrm{J}\mathrm{O}\mathrm{A}\;\mathrm{score}\right)\times 100/\left(17-\mathrm{Preop}\mathrm{erative}\;\mathrm{J}\mathrm{O}\mathrm{A}\;\mathrm{score}\right) $$

where postop. = postoperative, and preop. = preoperative.

The effects of therapy were classified into five grades: (1) excellent: recovery rate ≥80%; (2) good: recovery rate ≥50% but <80%; (3) effective: recovery rate ≥5% but <50%; (4) ineffective: recovery rate <5%; and (5) worse: symptoms were aggravated.

### Statistical analysis

The data are expressed as the mean ± standard deviation (SD). SPSS version 18.0 software (SPSS, Chicago, IL, USA) was used for the analysis. The *t* test was applied for comparisons between the two groups. Values of *p* < 0.05 were considered statistically significant.

## Results

The patients were followed up for 2.0 to 6.5 years after the operation (average 4.1 years). The operations of the two groups were performed smoothly, with mean operation durations of 2.5 and 2.1 h, respectively, and mean blood losses of 250 and 380 mL, respectively, in the anterior and posterior approach groups.

The anterior approach group experienced no aggravated neurological function or C5 nerve root palsy. Five patients had axial pain (relieved within 1 month), and two had temporary hoarseness (disappeared 3 months after operation). At 1 month postoperatively, radiography revealed titanium mesh subsidence in two patients and displacement of plates and screws in one. There was no further aggravation 3 months later. Bony fusion was achieved in all of the patients.

In contrast, in the posterior group, C5 nerve root palsy occurred in 2 patients (recovered 6 months postoperatively), axial pain in 15 patients (recovered within 1 month), and cerebrospinal fluid leakage in 3 patients (recovered within 3 weeks after a dressing change and other appropriate treatment). No instrumental failure or instability was observed (Table 1).

**Table 1:** **Surgical parameters and complications of patients who underwent an anterior approach or a posterior approach**

	**Anterior approach**	**Posterior approach**
Operation time (hour)	2.5	2.1
Blood loss (ml)	250	380
C5 palsy	0	2 (recovered in 6 months)
Axial pain	5 (recovered in 1 month)	15 (recovered in 1 month)
Hoarseness	2 (recovered in 3 months)	0
Cerebrospinal fluid leakage	0	3
Looseness of internal fixation	1 (stable in 3 months)	0
Bone healing	All	All

Radiography revealed that Cobb's angle was significantly improved postoperatively and during the follow-up in both groups compared with their preoperative values (*p* = 0.000). There was no significant difference in Cobb's angle between the two groups preoperatively (*p* = 0.567). The degree of improvement in the anterior approach group, however, was significantly higher than that in the posterior approach group, with statistically significant differences postoperatively and during the follow-up (*p* = 0.000) (Table 2).

**Table 2:** **Cobb's angle of the patients who underwent an anterior approach or a posterior approach**

	**Anterior approach**	**Posterior approach**
Preoperative	9.91 ± 7.51	10.41 ± 4.25^b^
Postoperative	22.27 ± 4.28^a^	15.97 ± 3.53^ac^
Follow-up	19.95 ± 4.27^a^	14.15 ± 3.72^ac^

^a^Compared with patients before operation in the same group, *p* < 0.05. ^b^Compared with anterior approach group, *p* > 0.05. ^c^Compared with anterior approach group, *p* < 0.05.

Our results also revealed that the JOA scores were significantly improved postoperatively and during the follow-up in the two groups compared with their preoperative values (*p* = 0.000). There were no significant differences in the JOA scores between the two groups preoperatively, postoperatively, or during the follow-up (*p* = 0.525). Postoperatively, the recovery rate was significantly higher for the anterior group than the posterior group (*p* = 0.028). In the anterior group, the recovery rate was evaluated as good in 30.23%, effective in 65.12%, and ineffective in 4.65% of patients postoperatively. At the last follow-up, the clinical outcome was excellent in 34.88% of patients, good in 60.47%, and effective in 4.65%. In the posterior group, the recovery rate was evaluated as good in 18.06% of patients, effective in 74.84%, ineffective in 6.45%, and worse in 0.65% postoperatively. At the last follow-up, the clinical outcome was excellent in 21.94% of patients, good in 61.94%, effective in 15.48%, and worse in 0.65%. Postoperatively and during the follow-up, the JOA scores recovery rate was significantly higher for the anterior group than for the posterior group (*p* < 0.05) (Table 3).

**Table 3:** **JOA scores of patients who underwent an anterior approach or a posterior approach**

	**Anterior approach**		**Posterior approach**	
	**JOA**	**Recovery rate**	**JOA**	**Recovery rate**
Preoperative	8.42 ± 2.30		8.62 ± 1.68^b^	
Postoperative	11.70 ± 2.21^a^	33.89 ± 17.44	10.97 ± 1.81^ab^	27.41 ± 19.17^c^
Follow-up	14.60 ± 1.50^a^	73.57 ± 14.00	14.22 ± 1.37^ab^	65.79 ± 17.22^c^

^a^Compared with patients before operation in the same group, *p* < 0.05. ^b^Compared with the anterior group, *p* > 0.05. ^c^Compared with the anterior group, *p* < 0.05.

## Discussion

Clinically, the basic principles for treating cervical degenerative diseases are to remove spinal cord compression, ensure recovery of the morphology of the spinal cord and vertebral canal volume, restore the cervical physiological curve, and achieve bony fusion. Posterior decompression surgery, including laminectomy and laminoplasty, has been used to allow the spinal cord to move backward, keeping it clear of anterior compression [10,11]. The magnitude of spinal posterior movement is limited, however, and excessive posterior movement of the spinal cord leads to tethering of the nerve root. The latter has been suggested to be the pathophysiological cause of segmental motor paresis (C5 nerve root palsy), which directly influences long-term postoperative outcomes [12]. In this study, two patients exhibited C5 nerve root palsy after undergoing posterior decompression. Additionally, the physiological curvature of the cervical spine cannot be restored using a posterior approach alone [13]. The loss of the physiological curvature and the emergence of kyphosis prior to the operation are contraindications to the posterior approach. Consequently, use of the posterior approach has been greatly limited.

The most common radiographic findings of multilevel cervical degenerative disease are (1) anterior compression resulting from osteophytes and hyperplasia of ligaments caused by disc herniation and (2) posterior compression mainly combined with hypertrophy of the ligamentum flavum. Patients with severe disease can develop ‘pincers’ symptoms, most of which are due to anterior compression. Thus, for the majority of the patients, an anterior approach is able to not only alleviate the compression but also to address mechanical problems.

An anterior approach that allows direct decompression has been more commonly used to treat cervical degenerative diseases. Nevertheless, many authors have thought that postoperative complications after multilevel anterior cervical corpectomy are more likely to develop with this approach [3,14-16]. It was previously reported that the nonunion rate for patients with a three-level corpectomy was 50%, compared with 9% for patients with a two-level corpectomy [17]. The high failure rate of multilevel corpectomy leads to the proposal of a posterior approach [3,14-16].

According to biomechanical research findings, a long plate, because of its long lever, can generate more flexibility under a physiological load at the fusion site [18,19]. Therefore, there is no decline in stability over the long term in patients who have undergone one-level corpectomy. Those with three-level standard corpectomy and internal fixation are more likely to develop failure of the internal fixation. For patients with four-level CSM, ‘skip’ corpectomy is a good alternative to avoid the multiple complications that appear after multilevel corpectomy [7]. According to our retrospective review, all patients who underwent ‘skip’ corpectomy achieved decompression. At 1-month postoperatively, radiography revealed titanium mesh subsidence in two patients and plate and screw dislodgement in one patient. Three months later, there was no further subsidence or dislodgement, and the bony grafts had fused.

Unlike multilevel corpectomy, ‘skip’ corpectomy for treating four-level CSM combines two one-level corpectomies to maintain the advantages of an anterior approach and eliminate the disadvantages of multilevel corpectomy. There are several advantages of anterior ‘skip’ corpectomy for multilevel CSM.

1. It offers direct decompression. Generally, multilevel CSM is caused by anterior compression, and an anterior approach can directly decompress the situation. Additionally, proper interbody distraction is performed during an anterior approach, so the posterior loosened ligamentum flavum can be tightened to eliminate the compression posterior to the spinal cord caused by the folds of ligamentum flavum, thereby improving the effectiveness of the decompression.
2. It restores the cervical physiological curvature. The patient's head is arched backward as required to restore the cervical physiological curvature as much as possible. When the distraction device is placed, the screw is perpendicular to the C5 vertebra, and the screws for C3 and C7 vertebrae are inclined cephalad and caudally as required to restore the C3 to C5 Cobb's angle and the C5 to C7 Cobb's angle, respectively (Figure 1A,B). After removing intervertebral discs, a distraction device is placed, and the anterior vertebral bodies are correspondingly distracted (restoring the cervical physiological curvature). A large visual area is needed to remove whatever is compressing the spinal cord, located in the posterior margin of the vertebrae. The removal can be performed under direct vision (Figure 1C). Finally, the plate is bent before placing it. The screw is first placed in the C3 and C7 vertebral bodies and then C5 to restore the cervical physiological curvature (Figure 1D,E).
3. It offers a relatively larger surgical field. ‘Skip’ corpectomy with a larger surgical field allows direct decompression at the level of the intervertebral space and posterior to the vertebral body. It can undercut decompression of the posterosuperior and posteroinferior aspects of the C5 vertebra (Figure 1B,C). Hence, this new technique can achieve complete decompression and be suitable to patients suffering from spinal canal stenosis of the C5 posterior margin and partial OPLL. Additionally, the possibility of damaging the cervical spinal cord can be reduced because the operation is performed under direct vision.
4. It increases stability. The ‘skip’ corpectomy eliminates the disadvantages of multilevel corpectomy, such as stress overconcentration. It preserves the C5 vertebra, thereby offering a plate with an intermediate attachment point and increasing the number of fixed screws to share stress. Thus, ‘skip’ corpectomy increases the postoperative stability of the cervical vertebrae.
5. It benefits bony fusion. The presence of cervical lordosis makes multilevel bone grafting difficult. Hence, by cutting down on the length of the graft and using two-level bone grafts for placement convenience, ‘skip’ corpectomy enlarges the host-graft interface and benefits postoperative bony fusion.

There were no significant differences in JOA scores reflecting clinical outcomes between the anterior and posterior approach groups preoperatively, postoperatively, or during follow-up. In contrast, the recovery rate of JOA scores and restoration of the cervical physiological curvature were significantly higher for the anterior approach group than for the posterior approach group. Restoration of the cervical physiological curvature obviously reduces the incidence of postoperative long-term complications.

Surprisingly, the number of complications in this study was low, and the fusion rate (100%) was high. One limitation in our research is that the number of cases was insufficient, which may have an influence on the conclusion. Additionally, the anterior approach group was too small to compare with the posterior approach group.

The optimal surgical approach for multilevel CSM or OPLL remains debatable. So long as surgical principles are strictly controlled, each approach (anterior, posterior, and combined) can be therapeutic with excellent efficacy. For restoring the cervical physiological curvature, however, an anterior approach may have better results than a posterior approach.

## Conclusions

‘Skip’ corpectomy displays safety comparable to that of posterior decompression and better efficacy for treating four-level cervical spondylotic myelopathy.
